# Development of A New Type of 2-DOF Piezo-Actuated Pseudo-Decoupled Compliant Mechanism for Elliptical Vibration Machining

**DOI:** 10.3390/mi10020122

**Published:** 2019-02-13

**Authors:** Rongqi Wang, Xiaoqin Zhou, Guangwei Meng

**Affiliations:** School of Mechanical and Aerospace Engineering, Jilin University, Changchun 130022, China; rqwang@jlu.edu.cn (R.W.); mgw@jlu.edu.cn (G.M.)

**Keywords:** compliant mechanism, CM, elliptical vibration machining, EVM) pseudo-decoupled, tracking accuracy, elliptical trajectory, flexure hinges

## Abstract

Currently, the elliptical vibration cutting/coining (EVC^2^) has been widely employed in fabricating various functional microstructure surfaces applied in many significant engineering fields. Therefore, for this study, a novel type of two-degree-of-freedom (2-DOF) piezoelectrically actuated pseudo-decoupled compliant mechanisms (PDCMs) with non-orthogonal decoupling structures, which can exactly generate the strict ellipse trajectories, was developed for improving the forming accuracies of the EVC^2^ microstructures. First, the compliance matrices of 2-DOF PDCMs were theoretically modeled using the popular finite beam-based matrix modeling (FBMM) and the matrix-based compliance modeling (MCM) methods, then finite element analysis (FEA) was adopted to verify the effectiveness of the built compliance model for the 2-DOF PDCM with arbitrary structure parameters. Second, the static FEA method was employed to systematically reveal the dependencies of the tracking accuracies of the elliptical trajectories on the decoupling structures of 2-DOF PDCMs. Moreover, their main dynamic performances were also investigated through the FEA-based harmonic response analysis and modal analysis. On these bases, the critical angle of the decoupling structure was optimally set at 102.5° so that the PDCMs had minimum shape distortions of the ellipse trajectories. Thirdly, a series of experiments was conducted on this PDCM system for practically investigating its kinematic and dynamic performances. The actual aspect ratio between the major axis and minor axis of the ellipse trajectory was approximately 1.057, and the first-order and second-order resonant frequencies were 863 Hz and 1893 Hz, respectively. However, the obtained testing results demonstrated well the effectiveness and feasibility of 2-DOF PDCM systems in precisely tracking the ellipse trajectories with different geometric parameters. Several critical conclusions on this study are summarized in detail in the final section of this paper.

## 1. Introduction

Elliptical vibration machining (EVM) has been extensively explored and applied in many significant fields, including the precise manufacturing of optical freeform surfaces and functional microstructure surfaces on various difficult-to-cut materials such as ferrous materials and carbide alloy [1,2,3,4,5,6]. Therefore, significant research efforts have been endlessly devoted to improving the working performances of EVM devices, such as increasing the resonant frequency, enhancing the motion accuracy, and optimizing the structure configurations. Based on these advantages of existing developments, the scale characteristics of these components fabricated by EVM can range from millimeter to micrometer and even to nanometer. In view of the EVM processes, both demand at least two high-frequency vibrations in the cutting-depth and up-feed directions, but the currently reported non-resonant EVMs with flexure-based mechanisms have rarely taken the cross-coupling motions across different vibrations into consideration [7,8], especially in those EVM systems that consist of flexural mechanisms with parallel configurations. As an adverse result, the parasitic distortions can be clearly observed on the elliptical trajectories generated by these EVM systems, which may further deteriorate the forming precision of the functional microstructures fabricated by the elliptical vibration cutting/coining (EVC^2^) [3,9]. It is therefore very important to innovatively develop a novel type of decoupled EVM systems for precisely manufacturing various functional microstructure surfaces.

Currently, a large number of piezoelectrically actuated compliant mechanisms (CMs) with two degrees of freedom (2-DOF) may be the potential substitutes for developing the much-needed EVM apparatuses. These 2-DOF CMs have already been very widely employed in optical and precision engineering, such as the XY stages for micro-/nanopositioning [10,11,12], the manipulators and grippers for micro-/nano-operation [13,14,15], and the fast tool servo (FTS) for micro-/nanomachining [16,17]. In general, various micro-/nanopositioning stages with parallel 2-DOF CMs can obtain high-precision motions through complex decoupling substructures. For example, Polit and Dong [18] have developed a parallel and decoupled XY positioning stage with a high working bandwidth for micro-/nanomachining by employing a novel hybrid compliant-notch parallelogram mechanism. However, the decoupling capacities of these XY stages will highly depend on the selections of dimensional parameters, thus it is not a universal method for achieving accurate motions [16]. In addition, almost all the developed XY position stages have generally adopted bisymmetric or serial configurations, but bisymmetric configurations will be difficult in compact structures and cutting tool installations, whereas serial configurations will decrease their working bandwidth because of increasing motion inertia [18,19,20]. In addition, the widely-used micro-/nanomanipulators and grippers have similar restrictions for decoupled EVC systems. The 2-DOF FTS can strictly track the elliptical trajectories for the EVC^2^ processes of functional microstructures due to their complete decoupling motions between two different motion directions, but it is very difficult to develop 2-DOF or multi-DOF parallel FTS mechanisms that can completely eliminate the cross-coupling motions across different directions, which will seriously restrict the application potentialities of FTS mechanisms in the significant EVC^2^ fields. 

To summarize, the reported micro-/nanopositioning XY stages, micro-/nanomanipulators and grippers cannot be directly applied to the EVC^2^ processes due to certain apparent limitations, and the FTS mechanisms are too rigorous to be widely applied to the EVC^2^ processes because of their development difficulties. Motivated by these potentials, this study aimed to develop a novel type of two-DOF piezo-driven pseudo-decoupled compliant mechanisms (PDCMs) to construct various elliptical vibration machining (EVM) systems by adopting the novel non-orthogonal decoupling substructures instead of the orthogonal configurations in traditional EVM mechanisms, which will greatly suppress or even eliminate the geometric distortions of elliptical trajectories. However, the developed 2-DOF PDCMs cannot completely decouple the parasitic motions in different directions although their tracking precision on elliptical trajectories can be greatly improved, so it is called a “pseudo-decoupled” PDCM. In view of the issues involved in the proposed 2-DOF PDCMs, the remainder of this paper is divided into several sections as follows. In Section 2, the compliance model of 2-DOF PDCM is theoretically constructed based on the popular matrix-based compliance modeling (MCM) and the powerful finite beam-based matrix modeling (FBMM) methods. Then, the feasibility and effectiveness of the built compliance model for 2-DOF PDCM is validated based on the finite element analysis (FEA) method. In Section 3, the decoupling characteristics as well as the static and dynamic performances of the 2-DOF PDCMs are also investigated using the static FEA method. In Section 4, we describe the series of testing experiments that were conducted on an established 2-DOF PDCM system for investigating its main kinematic and dynamic performances, involving the tracking precision and working bandwidth. Finally, several critical conclusions on current research are summarized in Section 5. 

## 2. Basic Principle and Compliance Model

**Figure 1:**
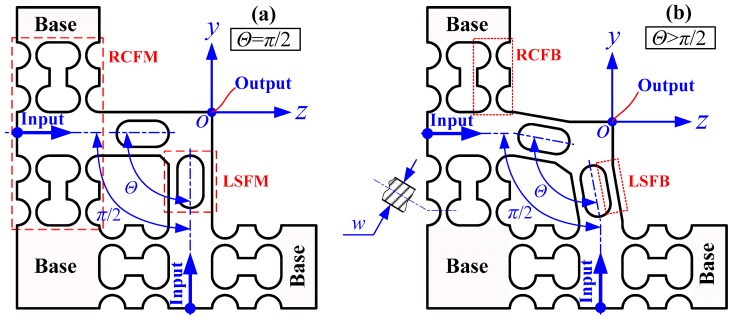
Basic principle of the developed two-degree-of-freedom (2-DOF) pseudo-decoupled compliant mechanism (PDCM). (**a**) Traditional orthogonal configuration; (**b**) Novel non-orthogonal configuration.

In the reported 2-DOF piezo-driven compliant mechanisms with decoupled motions, the bisymmetric CM structures, which have been parallelly composed of two typical flexure units shown in Figure 1a with central symmetric configurations, have extremely wide applications in the fields of micro-/nanopositioning, micro-/nanomanipulating and micro-/nanomachining. However, such types of bisymmetric CM structures are very difficult to integrate into elliptical vibration cutting/coining (EVC^2^) because of the redundant decoupling structures and hard cutting tool installations. Based on the above, the present study only adopted a typical flexure unit shown in Figure 1a to construct the required EVC^2^ systems, but the bad cross-decoupling motions of these asymmetric CM structures still had to be further eliminated to the best extent possible. Motivated by the reasons mentioned above, a novel type of piezo-driven 2-DOF pseudo-decoupled compliant mechanism (PDCM) was innovatively developed to precisely track the elliptical trajectories of EVC^2^ processes. It similarly employed two right circular flexure modules (RCFMs) composed of a group of parallel and symmetric right circular flexure hinges (RCFHs) as the driving and guiding units in two different directions. Two pairs of parallel filleted leaf-spring flexure hinges (LSFHs) were also adopted to construct two leaf-spring flexure modules (LSFMs) as the decoupling units in two different directions, as shown in Figure 1b. The innovation of this PDCM lies in that two decoupling LSFMs are non-perpendicularly configured with a decoupling angle Θ (i.e., Θ ≥ 90°) instead of the perpendicular configurations (i.e., Θ = 90°) that have been widely found in the traditional 2-DOF CMs for elliptical vibration machining (EVM). This novel type of PDCM cannot fully achieve the decoupling motions, although the elliptical trajectories can be strictly generated in practice, so it is called a “pseudo-decoupled” PDCM. 

### 2.1. The Principle of The FBMM Method

**Figure 2:**
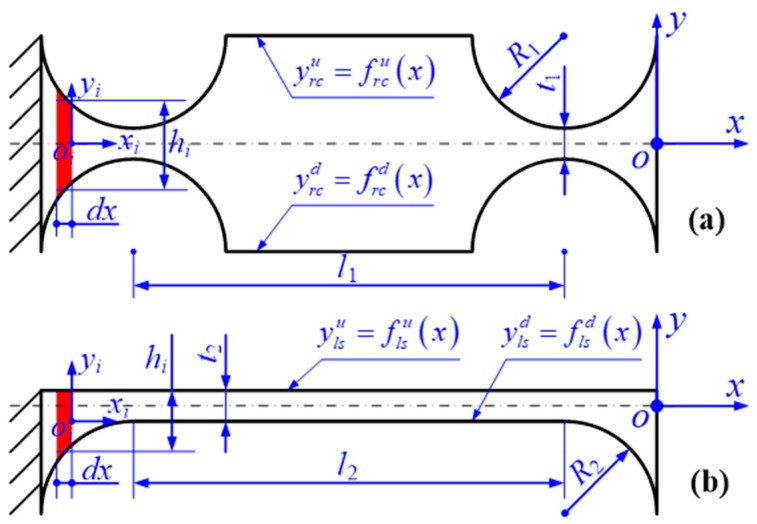
Schematic diagram of the finite beam-based matrix modeling (FBMM) method for two typical flexure beams. (**a**) Right circular flexure beam (RCFB); (**b**) Leaf-spring flexure beam (LSFB).

In order to analytically investigate the elastic deformation behaviors of the developed 2-DOF PDCMs, their compliance matrixes were mathematically modeled based on the popular matrix-based compliance modeling (MCM) method. As basic substructures, the elastic deformation behaviors of right circular flexure beams (RCFBs) and filleted leaf-spring flexure beams (LSFMs) must be first characterized by modeling their compliance matrixes. This paper introduced the powerful finite beam-based matrix modeling (FBMM) method developed by Zhu et al. and Wang et al. [21,22] to effectively construct the compliance models of RCFBs and LSFBs, respectively. The basic modeling principles are illustrated in Figure 2. 

For the FBMM processes, a flexure hinge was uniformly divided into *N* micro Euler–Bernoulli beams with different rectangular cross-sections, where *o_i_*-*x_i_y_i_z_i_* denotes the local Cartesian coordinate of the *i*-th micro-beam. In addition, we had to make the assumption that the effects of stress distributions on the elastic deformations of flexure beams could be ignored at first, and that each of the flexure hinges would be regarded as a series connection of all the micro-beams. Based on Hooke’s law, the relationship is first derived as follow:

(1) Δ=C F,

where **F** and **Δ** are defined as the unit load vector and the corresponding elastic deformation vector, respectively. **C** denotes the compliance matrix of the flexure hinge in the global coordinate, which can be mathematically expressed based on the matrix-based modeling (MCM) method [16,23] as follows:

(2) $$\begin{array}{cc} C=\sum_{i=1}^{N}T_{i}C_{i}T_{i}^{T}, & T_{i}=\left[\begin{array}{cc} R_{i} & S_{i}\left(r_{i}\right)R_{i}\\ 0 & R_{i} \end{array}\right] \end{array},$$

where **T***_i_* denotes the compliance transformation matrix (CTM) of the *i*-th local coordinate with respect to the global coordinate [12,13,23,24]. *N* is the total number of the divided micro-beams. **R***_i_* denotes the rotation matrix of the local coordinate with respect to the global coordinate. **r***_i_* represents the position vector of the local coordinate in the global coordinate. **S***_i_*(**r***_i_*) denotes the skew-symmetric operator for the position vector **r***_i_*.

(3) $$\begin{array}{cc} S_{i}\left(r_{i}\right)=\left[\begin{array}{ccc} 0 & -z_{i} & y_{i}\\ z_{i} & 0 & -x_{i}\\ -y_{i} & x_{i} & 0 \end{array}\right], & r_{i} \end{array}={\left[\begin{array}{c} x_{i}\\ y_{i}\\ z_{i} \end{array}\right]}^{T}={\left[\begin{array}{c} dx\cdot i\\ f^{u}\left(x_{i}\right)/2+f^{d}\left(x_{i}\right)/2\\ 0 \end{array}\right]}^{T}$$

**C***_i_* denotes the compliance matrix of the *i*-th single micro-beam in its local coordinate, which has been proposed and widely employed in characterizing the elastic deformation behaviors of leaf-spring flexure hinges [24,25], as mathematically expressed by the equation below:

(4) $$C_{i}=\left[\begin{array}{cccccc} \frac{dx}{Ewh_{i}} & 0 & 0 & 0 & 0 & 0\\ 0 & \frac{4dx^{3}}{Ewh_{i}{}^{3}}+\frac{\alpha_{s}dx}{Gwh_{i}} & 0 & 0 & 0 & \frac{6dx^{2}}{Ewh_{i}^{3}}\\ 0 & 0 & \frac{4dx^{3}}{Ew^{3}h_{i}}+\frac{\alpha dx}{Gwh_{i}} & 0 & -\frac{6dx^{2}}{Ew^{3}h_{i}} & 0\\ 0 & 0 & 0 & \frac{dx}{Gkwh_{i}^{3}} & 0 & 0\\ 0 & 0 & -\frac{6dx^{2}}{Ew^{3}h_{i}} & 0 & \frac{12dx}{Ew^{3}h_{i}} & 0\\ 0 & \frac{6dx^{2}}{Ewh_{i}^{3}} & 0 & 0 & 0 & \frac{12dx}{Ewh_{i}^{3}} \end{array}\right].$$

In Equation (4), *E* and *G* are the modulus of elasticity and the modulus of rigidity. *dx*, *w*, and *h_i_* are the length, width, and thickness of the *i*-th micro-beam, *h_i_* is obtained by the formulations *h_i_*(*x*) = |*y_i_^u^* − *y_i_^d^*| = | *f ^u^*(*x_i_*) − *f ^d^*(*x_i_*)|. *k* is the shape factor of torsional deformation [24], which is defined as *k*=*w*/*h*. With the Poisson ratio *μ*, the shear coefficient *α_s_* was introduced for the micro-beams with rectangular cross-sections [26].

(5) $$\alpha_{s}=\frac{12+11\mu }{10(1+\mu )}$$

In addition, based on the definition of this ratio *z_i_* = *w_i_*/*h_i_*, a new torsion compliance, which is independent on the ratio of width to the thickness, was adopted to more accurately characterize the torsion behavior *θ_x_*/*M_x_* of the *i*-th micro-beam [27].

(6) $$C_{\theta_{x},M_{x}}^{i}=\frac{7dx}{2G}\left(\frac{1}{w_{i}b_{i}{}^{3}}+\frac{1}{w_{i}{}^{3}b_{i}}\right)\frac{z_{i}{}^{2}+2.609z_{i}+1}{1.17z_{i}{}^{2}+2.191z_{i}+1.17}$$

Finally, the notch-shape functions *f*_rc_(•) and *f*_ls_(•) of the RCFB and LSFB were geometrically defined, then their compliance matrix **C**_rcfb_ and **C**_lsfb_ was mathematically formulated as follows:

(7) $$C_{\mathrm{rcfb}}=\sum_{i=1}^{N}T_{i}C_{i}T_{i}^{T}|\begin{array}{l} f_{rc}^{u}\left(i\cdot dx\right)\\ f_{rc}^{d}\left(i\cdot dx\right) \end{array},$$

(8) {frcu(x)=t12+R1−R12−(x+l1+R1)2,|x+l1+R1|<R1frcu(x)=t12+R1,−l1≤x≤−2R1frcu(x)=t12+R1−R12−(x+R1)2,|x+R1|<R1;frcd(x)=−frcu(x),

(9) $$C_{\mathrm{lsfb}}=\sum_{i=1}^{N}T_{i}C_{i}T_{i}^{T}|\begin{array}{l} f_{\mathrm{ls}}^{u}\left(i\cdot dx\right)\\ f_{\mathrm{ls}}^{d}\left(i\cdot dx\right) \end{array},$$

(10) {flsd(x)=R22−(x+l2+R2)2−t22−R2,−l2−2R2≤x<−l2−R2flsd(x)=−t22,−l1−R2≤x≤−R2flsd(x)=R22−(x+R2)2−t22−R2,−R2<x≤0;flsu(x)=t22.

### 2.2. The Compliance Model of The 2-DOF PDCM

**Figure 3:**
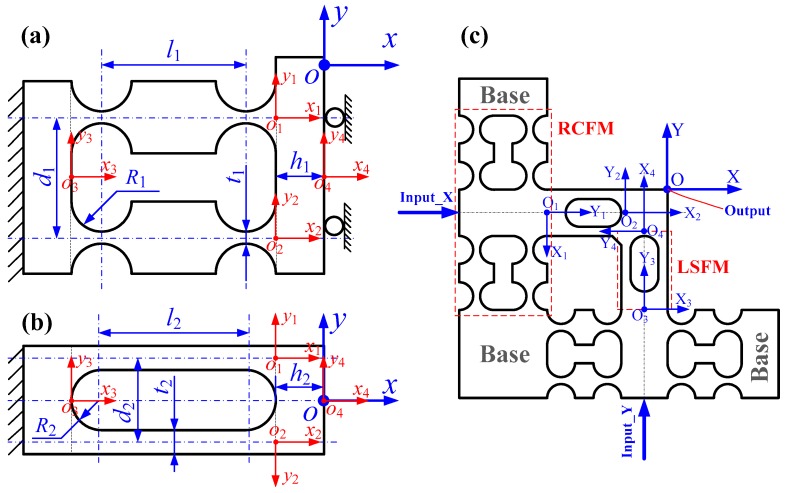
Schematic diagram of the MCM method for the 2-DOF pseudo-decoupled compliant mechanism (PDCM). (**a**) Right circular flexure module (RCFM), (**b**) Leaf-spring flexure module (LSFM), (**c**) 2-DOF PDCM.

Based on the popular RCFBs and LSFBs mentioned above, the right circular flexure modules (RCFMs) and leaf-spring flexure modules (RCFMs) were constructed to develop a type of 2-DOF pseudo-decoupled compliant mechanisms (PDCM), as shown in Figure 3. In order to understand the basic attributes of the flexural mechanisms, the matrix-based compliance modeling (MCM) method was adopted to model the compliances of the 2-DOF PDCM for analytically characterizing its elastic deformation behaviors. First, the driving and guiding RCFM was constructed through the parallel connection of four identical RCFBs with perfectly symmetrical configurations. Its structural features and the dimensional parameters are illustrated in Figure 3a. Based on the MCM method, the compliance matrix of the adopted RCFM can be mathematically expressed by the following:

(11) $$C_{\mathrm{rcfm}}={\left[{\left(C_{\mathrm{rcfm}}^{Right}\right)}^{-1}+{\left(C_{\mathrm{rcfm}}^{Left}\right)}^{-1}\right]}^{-1},$$

(12) $$C_{\mathrm{rcfm}}^{Right}=T_{\mathrm{rcfm}}^{3}C_{L}^{3}{\left(T_{\mathrm{rcfm}}^{3}\right)}^{T}+{\left\{{\left[T_{\mathrm{rcfm}}^{1}C_{\mathrm{rcfb}}^{1}{\left(T_{\mathrm{rcfm}}^{1}\right)}^{T}\right]}^{-1}+{\left[T_{\mathrm{rcfm}}^{2}C_{\mathrm{rcfb}}^{2}{\left(T_{\mathrm{rcfm}}^{2}\right)}^{T}\right]}^{-1}\right\}}^{-1}+T_{\mathrm{rcfm}}^{4}C_{L}^{4}{\left(T_{\mathrm{rcfm}}^{4}\right)}^{T},$$

where **C**_L_ represents the basic compliance matrix of the LSFHs in its local coordinate and $T_{\mathrm{rcfm}}^{i}$ (*i* = 1, 2, 3, 4) denotes the compliance transformation matrix (CTM) of the *i*-th flexure structure in the global coordinate of the RCFM. Based on the structure symmetry, the compliances of the left half can be simply obtained by rotating the compliance of the right half, which can be mathematically derived by the following equation:

(13) $$C_{\mathrm{rcfm}}^{Left}=T_{ry}\left(\pi \right)C_{\mathrm{rcfm}}^{Right}T_{ry}{\left(\pi \right)}^{T},$$

where **T***_ry_*(π) denotes the rotation transformation matrix around the *y*-axis with an angle of *π*. 

Second, the decoupling LSFM was constructed through the parallel connection of two LSFBs, as shown in Figure 3b. The compliance matrix of the LSFM can be mathematically expressed as follows:

(14) $$C_{\mathrm{lsfm}}=T_{\mathrm{lsfm}}^{3}C_{L}^{3}{\left(T_{\mathrm{lsfm}}^{3}\right)}^{T}+{\left\{{\left[T_{\mathrm{lsfm}}^{1}C_{\mathrm{lsfb}}^{1}{\left(T_{\mathrm{lsfm}}^{1}\right)}^{T}\right]}^{-1}+{\left[T_{\mathrm{lsfm}}^{2}C_{\mathrm{lsfb}}^{2}{\left(T_{\mathrm{lsfm}}^{2}\right)}^{T}\right]}^{-1}\right\}}^{-1}+T_{\mathrm{lsfm}}^{4}C_{L}^{4}{\left(T_{\mathrm{lsfm}}^{4}\right)}^{T},$$

where $T_{\mathrm{lsfm}}^{i}$ (*i* = 1, 2, 3, 4) denotes the CTM of the *i*-th flexure structure with respect to the global coordinate of the LSFM. Finally, a single-DOF compliant mechanism was constructed through the series connection of an RCFM and an LSFM, then two such single-DOF compliant mechanisms were parallelly and non-orthogonally connected to construct the developed 2-DOF PDCM, as illustrated in Figure 3c. Based on the MCM method, the compliance model of this PDCM can be mathematically formulated as follows:

(15) $$C_{\mathrm{pdcm}}={\left\{{\left[T_{\mathrm{pdcm}}^{1}C_{\mathrm{rcfm}}^{1}{\left(T_{\mathrm{pdcm}}^{1}\right)}^{T}+T_{\mathrm{pdcm}}^{2}C_{\mathrm{lsfm}}^{2}{\left(T_{\mathrm{pdcm}}^{2}\right)}^{T}\right]}^{-1}+{\left[T_{\mathrm{pdcm}}^{3}C_{\mathrm{rcfm}}^{3}{\left(T_{\mathrm{pdcm}}^{3}\right)}^{T}+T_{\mathrm{pdcm}}^{4}C_{\mathrm{lsfm}}^{4}{\left(T_{\mathrm{pdcm}}^{4}\right)}^{T}\right]}^{-1}\right\}}^{-1},$$

where $T_{\mathrm{pdcm}}^{i}$ (*i* = 1, 2, 3, 4) denotes the CTM of the *i*-th flexure module with respect to the global coordinate of the developed 2-DOF PDCM. 

## 3. Finite Element Analysis of The 2-DOF PDCM

### 3.1. FEA Verification

At this stage, the effectiveness of the built compliance model must first be verified, and a series of FEA processes was therefore conducted on the 2-DOF PDCM with an arbitrary set of dimension parameters, as listed in Table 1. Then, the compliances of dominant motions and parasitic motions, namely, *dx*/*Fx* and *dx*/*Fy* or *dy*/*Fy* and *dy*/*Fx*, were obtained, as listed in Table 1. In addition, the FEA results were considered the “accurate” values to evaluate the prediction accuracy of the built compliance model, and the relative deviations between the analytical results and FEA results were further calculated. From the comparative analysis listed in Table 1, the relative deviations between the FEA results and the analytical results in the compliances *dx*/*Fx* and *dx*/*Fy* are approximately 8.7% and 5.2%, respectively, which distinctly indicates that the compliance model can effectively characterize the elastic deformation behaviors of the developed 2-DOF PDCM. However, the relatively large error may stem mainly from the complex shear effects and stress concentration effects in the connections of the RCFMs and LSFMs. 

### 3.2. Decoupling Analysis

**Figure 4:**
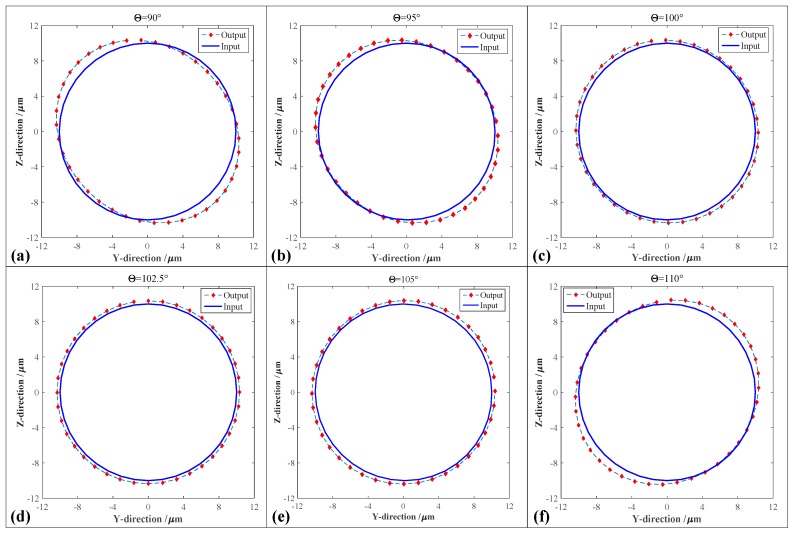
Comparative analysis of the elliptical trajectories of the 2-DOF PDCM with different decoupling angles Θ. (**a**) Θ = 90°; (**b**) Θ = 95°; (**c**) Θ = 100°; (**d**) Θ = 102.5°; (**e**) Θ=105°; (**f**) Θ = 110°.

**Figure 5:**
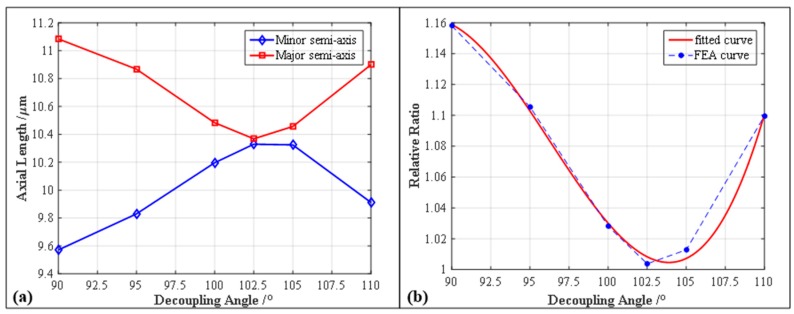
The influences of the PDCM’s decoupling angle Θ on the elliptical parameters and relative ratio. (**a**) Axial length *a* and *b* vs. decoupling angle Θ; (**b**) Aspect ratio *λ* vs. decoupling angle Θ.

Based on the above-mentioned built compliance model, the traditional 2-DOF PDCM with the orthogonal decoupling configuration was analytically constructed to minimize the cross-coupling motions by optimally determining the structure parameters of RCFMs and LSFMs. Subsequently, another type of 2-DOF PDCM with an innovative non-orthogonal configuration was developed to generate pseudo-decoupled motions for the widely-used elliptical vibration cutting/coining (EVC^2^), but the crucial decoupling angle Θ of the non-orthogonal configuration had uncertain influences on the tracking precision of elliptical trajectories at the time. Therefore, this study investigated the influences of the two-DOF PDCMs’ decoupling angle Θ on the tracking accuracies of elliptical trajectories based on the static FEA method, and further determined the optimal decoupling angle Θ. In addition to a greater operational optimization of the decoupling angle, this study introduced a dimensionless aspect ratio (DAR) *λ* of the major semi-axis *a* to the minor semi-axis *b* of the ellipse trajectory. All the major semi-axes of the elliptical trajectories are specifically equal to the minor semi-axes, namely *λ = a/b =*1. Then the results obtained from the FEA-based decoupling analysis are shown in Figure 4 and Figure 5. 

In the FEA investigations about the effects of the decoupling angle Θ, two equal-amplitude harmonic motions with a 90° phase difference were respectively employed in driving the Y-axis and Z-axis of the 2-DOF PDCMs with different decoupling angles Θ, thereby making the input elliptical trajectories perfectly circular. Driven by the circular trajectories, the 2-DOF PDCMs with different decoupling angles generated the elliptical trajectories with different elliptical parameters based on the FEA method, as illustrated in Figure 4. Subsequently, in order to quantitatively evaluate the decoupling capacities of the developed 2-DOF PDCMs, the corresponding elliptical trajectories whose decoupling angles Θ ranged from 90° to 110° were mathematically fitted with the least square method (LSM). Then the influences of the decoupling angle Θ on the geometric parameters of the elliptical trajectories were analytically investigated, involving the major semi-axis *a*, minor semi-axis *b* and aspect ratio *λ*, as shown in Figure 5. The dependency of the aspect ratio *λ* on the decoupling angles Θ was also fitted with a three-order polynomial. 

As shown in Figure 4a, the traditional 2-DOF PDCM with orthogonal configuration (Θ = 90°) had an obvious geometric distortion on its output trajectory. However, the trajectory distortions of the innovative 2-DOF PDCMs with non-orthogonal configurations gradually decreased when the decoupling angle Θ ranged from 90° to 102.5°, as shown in Figure 4b–d. Meanwhile, the trajectory distortions of the 2-DOF PDCMs gradually increased when the decoupling angles Θ were greater than 102.5°, but the inclined angles of the output ellipse trajectories had opposite directions with the ellipse trajectories whose decoupling angles were less than 102.5°, as shown in Figure 4d–f. From the fitted results shown in Figure 5, the major semi-axes *a* of the ellipse trajectories had gradually decreasing lengths when the decoupling angle Θ increasingly ranged from 90° to 102.5°, whereas the lengths of the major semi-axes *a* gradually increased when the decoupling angles Θ exceeded 102.5°. The minor semi-axes *b* of the ellipse trajectories had contrary changing laws with the major semi-axes *a*, but the major and minor semi-axes had respectively the minimum and maximum lengths when Θ was 102.5°. Similarly, the defined aspect ratio *λ* of the ellipse trajectories gradually decreased when the range of the decoupling angle Θ increased from 90° to 102.5°, but increased with the increasing angle Θ (Θ > 102.5°), as shown in Figure 5b. It is clear that the dimensionless aspect ratio *λ* also had the minimum value (1.003756) when Θ = 102.5°, which is very close to the perfect DAR (*λ* = 1). To summarize, the developed 2-DOF PDCM generated an approximately perfect ellipse trajectory when its decoupling angle was optimally chosen to be 102.5°. Of particular note is that the output trajectories of the 2-DOF PDCM with different decoupling angles were slightly larger than the input trajectories, as shown in Figure 5, which indicates that the 2-DOF PDCMs can definitely amplify the input motions. This provides an interesting potential to develop other compliant mechanisms (CMs) in those fields that urgently require large displacements. 

**Figure 6:**
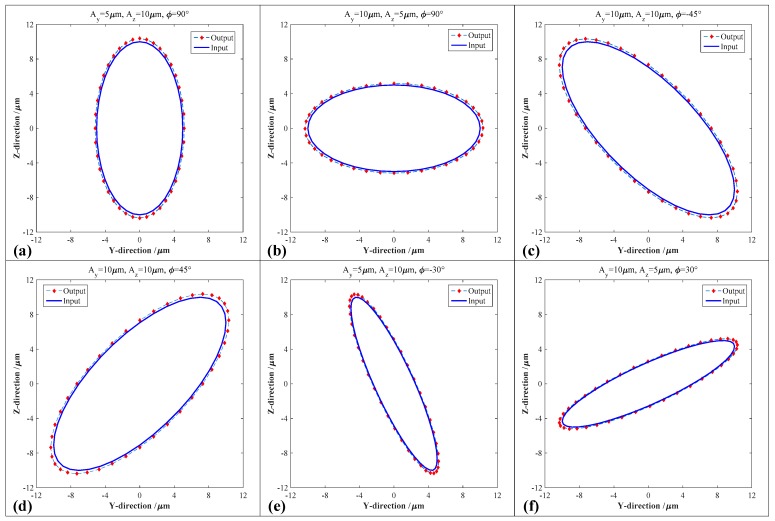
Comparative analysis of the elliptical trajectories generated by the 2-DOF PDCM under different elliptical parameters. (**a**) *A_y_* = 5 μm, *A_z_* = 10 μm, *φ* = 90°; (**b**) *A_y_* = 10 μm, *A_z_* = 5 μm, *φ* = 90°; (**c**) *A_y_* = 10 μm, *A_z_* = 10 μm, *φ* = −45°; (**d**) *A_y_* = 10 μm, *A_z_* = 10 μm, *φ* = 45°; (**e**) *A_y_* = 5 μm, *A_z_* = 10 μm, *φ* = −30°; (**f**) *A_y_* = 10 μm, *A_z_* = 5 μm, *φ* = 30°.

However, all the FEA-based investigations, which were conducted on the 2-DOF PDCM with different decoupling angles, completely adopted the circular trajectories to expediently verify their decoupling performances. The decoupling performances of the 2-DOF PDCM with the optimal decoupling angle Θ (102.5°) on the ellipse trajectories must be further discussed in detail. Therefore, the input motions with different elliptical parameters were similarly employed in analyzing the geometric distortions of the corresponding ellipse trajectories based on the FEA method. The results obtained under different vibration amplitudes and phase differences are illustrated in Figure 6. More specifically, Figure 6a,b shows the elliptical trajectories with different vibration amplitudes in the Y-axis and Z-axis, respectively, Figure 6c,d shows the elliptical trajectories with different phase differences, and Figure 6e,f shows the elliptical trajectories with both different vibration amplitudes and phase differences. Almost all the output ellipse trajectories can strictly track the input motions except for the slight geometric expansions, and their elliptical distortions along the major axis are much larger than those along the minor axis. However, all the resulted ellipse trajectories distinctly indicate that the developed 2-DOF PDCM with the optimal configuration can precisely generate the pseudo-decoupled elliptical trajectories with different parameters, which proves the effectiveness and feasibility of this type of developed 2-DOF PDCM in constructing various elliptical vibration cutting/coining (EVC^2^) apparatuses that have been widely employed in the manufacture of many functional microstructure surfaces.

### 3.3. Static and Dynamic Analysis

**Figure 7:**
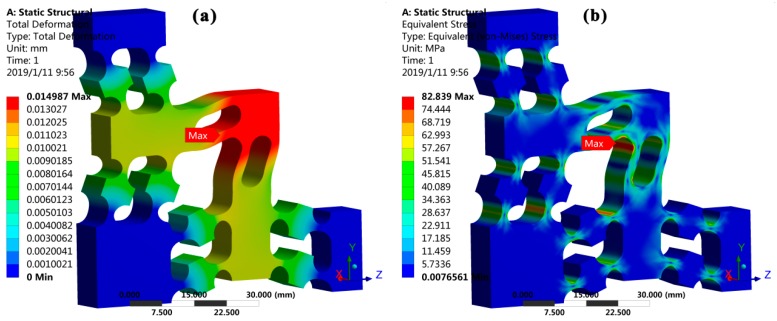
The static analysis of the developed 2-DOF PDCM. (**a**) Displacement nephogram and (**b**) stress nephogram.

Except for the tracking accuracy of the elliptical trajectory, the developed 2-DOF PDCM also ensured its maximum stress within the allowable value of material strength. Considering the chosen piezoelectric stack (PZT) actuators (P-887.51) with a stiffness of 100 N/μm and a stroke of 15 μm, as well as their preloading effects and the stiffnesses of the PDCM, the estimated maximum displacements of the PDCM’s input-ends were both chosen as 10 μm in the Y and Z directions. As a consequence, the total displacement and von Mises stress of the 2-DOF PDCM was simulated based on the static FEA method, and then the corresponding displacement nephogram and stress nephogram were obtained, respectively, as shown in Figure 7. 

From the displacement nephogram shown in Figure 7a, the maximum output displacement of the PDCM is approximately 14.99 μm when its input-ends in the Y and Z directions are both driven with PZT maximum strokes of 10 μm. At that time, the corresponding maximum stress of the PDCM is approximately 82.84 MPa, which is located at the intersection of the two decoupling LSFMs, a value much less than the allowable strength of the chosen material (spring steel 65Mn) for the PDCM. However, the FEA results clearly suggest that the 2-DOF PDCM can completely guarantee the elastic deformations with long-term linearity and repeatability. 

**Figure 8:**
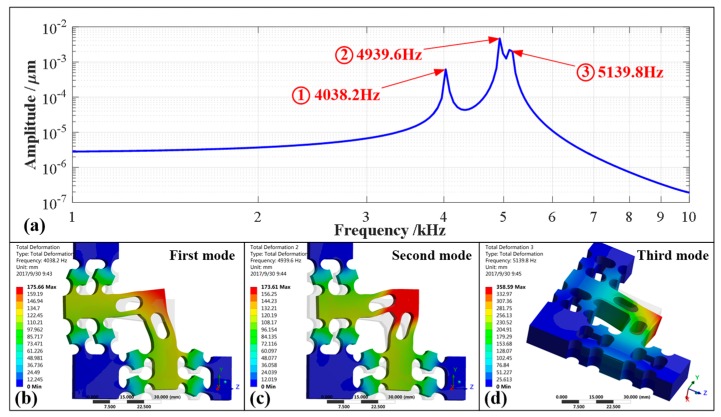
Dynamic study of the 2-DOF PDCM based on the finite element analysis (FEA) method. (**a**) Harmonic analysis; (**b**) First mode (4038.2 Hz); (**c**) Second mode (4939.6 Hz); (**d**) Third mode (5139.8 Hz).

Moreover, dynamic performances such as the working bandwidth were also very important for this developed 2-DOF PDCM, especially for the EVC^2^ processes that require that the elliptical vibrations have the highest available frequencies. Therefore, a harmonic response analysis and a modal analysis had to be further conducted to investigate the dynamic performances of the optimal PDCM using a commercial FEA software, respectively. Subsequently, the amplitude-frequency characteristics and the modal shapes of the first three vibrations were obtained, as illustrated in Figure 8. From the resulting harmonic analysis shown in Figure 8a, the resonant frequencies of the PDCM from the first order to the third order are 4038.2 Hz, 4939.6 Hz, and 5139.8 Hz, respectively. The vibrations of the output platform in the elliptical trajectory plane (*Y*-*O*-*Z* plane) are found in the first-order and second-order models shown in Figure 8b,c, respectively, but the vibrations of the output platform in the *Y*-*O*-*Z* plane occur in the third-order mode shown in Figure 8d. Fortunately, the second-order and third-order frequencies were much higher than the first-order frequency, and the first-order mode was thus the most prominent mode, which strongly determined the available working bandwidth of the PDCM system. 

**Figure 9:**
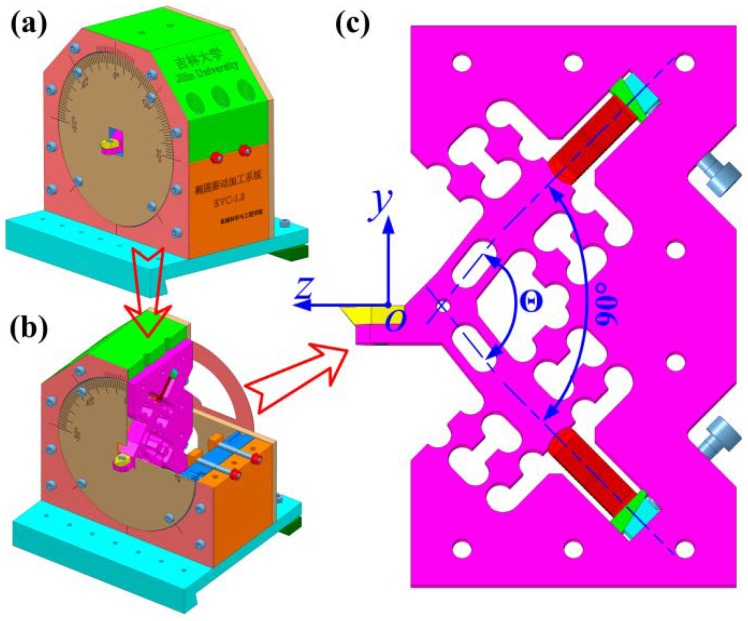
The 3D model of the (**a**) EVC system and (**b**) its sectional view, as well as the (**c**) modified 2-DOF PDCM.

Eventually, the resulting decoupling investigations, static analyses, and dynamic analyses distinctly indicated that the developed 2-DOF PDCM possessed a high effectiveness and a strong feasibility. However, it was still difficult to directly construct a usable EVC^2^ apparatus because no consideration had been given to the advantages of installing it on a machine tool, the installations and preloads of two PZT actuators, as well as the structure interference between the EVC^2^ system and the lathe spindle. Therefore, the 2-DOF PDCM was further modified by performing a 90° rotation and adding preload structures with wedge blocks, as shown in Figure 9. No more explanations are given here due to the limited time and space. 

## 4. Experimental Verification

**Figure 10:**
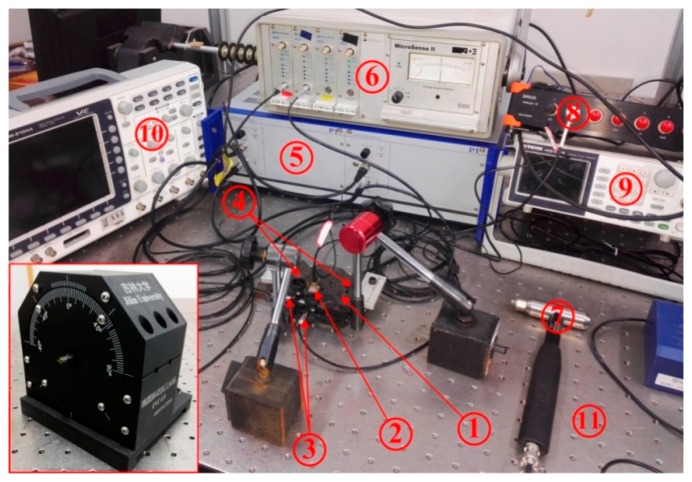
Photographic representation of the experimental setup for the 2-DOF PDCM system. 1-Compliant mechanism; 2-Acceleration transducer; 3-Probes of the capacity transducers; 4-Piezoelectric stack actuators; 5-Power amplifier; 6-Multi-channel position measuring module; 7-Impact hammer; 8-High speed collecting and analyzing module; 9-Signal generator; 10-Oscillograph; 11-Air-bearing vibration-isolated platform.

In order to verify the effectiveness and feasibility of the modeling compliance and optimizing structure that were conducted on the proposed 2-DOF pseudo-decoupled compliant mechanisms (PDCMs), their actual trajectory precision and working bandwidths had to be also experimentally investigated, respectively. A photographic representation of the experimental system’s setup is shown in Figure 10. First, two piezoelectric stack (PZT) actuators (P-887.51, PI, Karlsruhe, Germany) were employed in driving the 2-DOF PDCM in the two different directions, and two low-frequency harmonic signals with same voltage amplitudes and a phase difference were generated through a programmable signal generator (AFG-2225, Gwinstek, Taiwan, China) to respectively excite these two adopted PZT actuators. Subsequently, both the real-time displacements of the PDCM in two different directions were measured practically through two capacitive probes (2805, MicroSense, Lowell, MA, USA) and a multi-channel position measuring module (Model 5300 II, MicroSense, Lowell, MA, USA), which were employed for the experimental investigations about the tracking accuracy of the 2-DOF PDCM’s elliptical trajectory. It is very important to note that the amplitudes of the two adopted driving voltages had to be slightly adjusted for exactly controlling the same motion amplitudes in the Y and Z directions. This was mainly due to the differences in the two PZT preloads in the Y and Z directions, which resulted in the different motion amplitudes under the same driving voltages. Moreover, the manufacturing defects of the PDCM had adverse influences. In addition, all the experimental tests were conducted on an air-bearing vibration-isolated platform (RS4000, Newport Corporation, Irvine, CA, USA) for minimizing the adverse effects of external environmental disturbances.

### 4.1. Kinematic Performance

**Figure 11:**
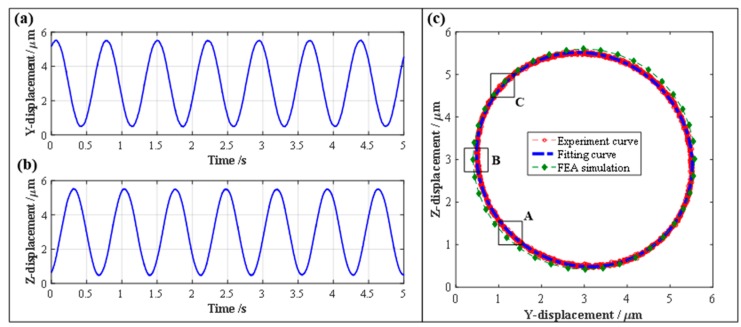
The experimental results of kinematic performance of the 2-DOF PDCM system. (**a**) Displacement in Y-axis, (**b**) Displacement in Z-axis, (**c**) Elliptical trajectory.

In view of the actual performance characteristics of the adopted PZT actuators, the vibration amplitudes and center offsets of the PDCM’s elliptical trajectory both in the Y and Z directions were theoretically chosen as 2.5 μm and 3 μm, respectively. The output displacements in these two different directions and the elliptical trajectory could then be generated practically through the use of two harmonic voltage signals that possessed suitable amplitudes, center offsets and phase differences, as shown in Figure 11. Subsequently, both the output displacements and the elliptical trajectories were mathematically fitted with the least square method (LSM), their corresponding fitting parameters are listed in Table 2. 

As shown in Figure 11a,b, the motion amplitudes of the output displacements in the Y and Z directions are 2.5151 μm and 2.5132 μm, respectively, and the center offsets in the Y and Z directions are 3.0000 μm and 2.9990 μm, which are very close to the preset amplitude (2.5 μm) and offset (3.0 μm). Then, the elliptical trajectories were generated practically and mathematically fitted, and the FEA-based elliptical trajectory was simulated to further conduct comparative investigations on the experimental and fitting trajectories, as shown in Figure 11c. From the fitted experimental results listed in Table 2, the motion amplitudes and center offsets of the experimental trajectory in the Y and Z directions are 2.5841 μm, 2.4449 μm and 3.0012 μm, 2.9986 μm, respectively. It is very clear that the experimental trajectory is very close to the FEA-based ellipse trajectory. However, the relative aspect ratio *λ* of the generated and simulated elliptical trajectories are 1.057 and 1.004, which is due to the PDCM’s manufacturing defects as well as the fact that the assembly errors and preload differences of the PZT actuators may have distorted the experimental ellipse trajectory of the 2-DOF PDCM system. Notwithstanding the above, the aspect ratio *λ* of the experimental ellipse trajectory is very close to its perfect value (*λ* = 1), which clearly indicates the effectiveness and feasibility of the established 2-DOF PDCM system in precisely tracking the strict ellipse trajectories. 

**Figure 12:**
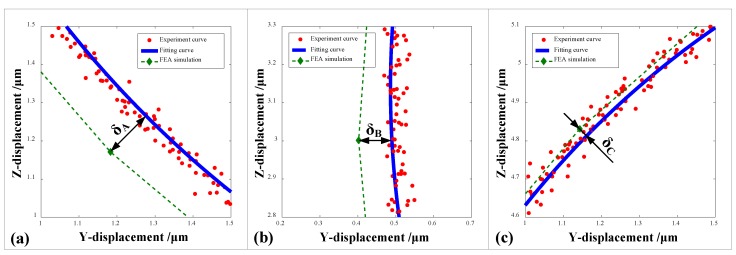
Comparative analysis of the tracking precisions of local elliptical trajectories generated by the 2-DOF PDCM. (**a**) Region A, *δ*_A_ = 0.1331 μm; (**b**) Region B, *δ*_B_ = 0.0873 μm; (**c**) Region C, *δ*_C_ = 0.0256 μm.

The established 2-DOF PDCM system can effectively and strictly generate the elliptical trajectories, but certain tracking errors were still found between the experimental and FEA results shown in Figure 11c. Therefore, this study further conducted error analyses on three local ellipse trajectories for the 2-DOF PDCM, namely, the regions marked by A, B, and C and shown in Figure 11c. Then, the resulting tracking errors in the different regions were mathematically calculated, as shown in Figure 12. It is clear that the maximum and minimum tracking errors of the elliptical trajectories are found in regions A and C. The tracking deviations for the regions A, B, and C are *δ*_A_ = 0.1331 μm, *δ*_B_ = 0.0873 μm, and *δ*_C_ = 0.0256 μm, respectively, whose corresponding ratios (i.e., relative errors) to their semi-major axis length of the elliptical trajectory are 5.3%, 3.5%, and 1.0%, respectively. This clearly indicates that the established 2-DOF PDCM system can greatly satisfy the tracking precision of elliptical trajectories. However, in examining all the experimental and FEA-based elliptical trajectories shown in Figure 11c, it is of particular note that the resulting FEA-based ellipse is slightly larger than the experimental ellipse at different levels. This is mainly due to the fact that the 2-DOF PDCMs slightly amplified the input displacements both in the Y and Z directions, as illustrated in Figure 4 and Figure 6. 

### 4.2. Dynamic Performance

**Figure 13:**
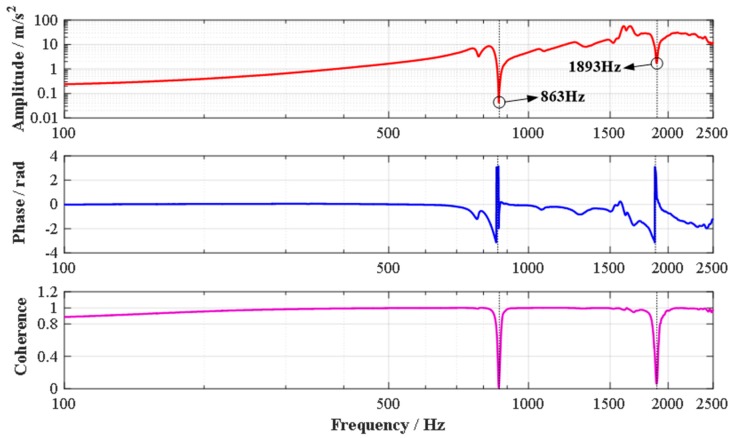
The experimental results of the dynamic performances of the 2-DOF PDCM system. (**a**) Amplitude-frequency; (**b**) Phase-frequency; (**c**) Coherence-frequency.

Similarly, in order to investigate the dynamic performances of the established 2-DOF PDCM system, the working bandwidth was experimentally measured based on the hammering method. Firstly, an acceleration transducer (ULT2001, Lancetec, Akron, OH, USA) was installed on the output platform of the 2-DOF PDCM to measure the real-time acceleration values as response signals when the PDCM was directionally stroked with an impact hammer (9724A2000, Kistler, Winterthur, Switzerland). Secondly, both the acceleration signals of the transducer and the force spectrums of the impact hammer were collected in real time with a high-speed collecting and analyzing module (SIRIUS i-HS 8×ACC, DEWEsoft, Kumberg, Austria). Finally, the vibration amplitude, phase, and coherence of the hammering experiments on the PDCM system were effectively obtained using an established dynamic testing system and a commercial modal analysis software (DEWEsoft X2, Kumberg, Austria), as shown in Figure 13. 

From the experimental results shown in Figure 13, many wave peaks/valleys were observably found both in the amplitude-frequency and phase-frequency charts, which may indicate different order resonant frequencies of the built 2-DOF PDCM system. However, two wave peaks/valleys were the most evident when the corresponding frequencies were 863 Hz and 1893 Hz., In addition, only two sharp changes can be clearly observed in the coherence-frequency charts at the above two critical frequencies. The actual first-order and second-order resonant frequencies of this established 2-DOF PDCM system can therefore be synthetically determined as 863 Hz and 1893 Hz. Moreover, we can confidently conclude that the coherence-frequency curve is perfectly close to 1 except for the two points near the first-order and second-order resonant frequencies, which demonstrates well the effectiveness of the dynamic experiments conducted on the PDCM system. However, the actual first-order and second-order resonant frequencies of the PDCM system are 863 Hz and 1893 Hz, respectively, which are much lower than the FEA-based simulated results (4038.2 Hz and 4939.6 Hz). This is mainly because of two reasons: (a) the imperfect contacts between the PDCM and PZT actuators; (b) the increases in moving inertia induced by the adopted acceleration transducer with 8gm mass that is close to the output platform mass of the PDCM. These are therefore the main factors of the deteriorating working bandwidth.

## 5. Conclusions

In order to simply but strictly track the ellipse trajectories that have been widely applied in elliptical vibration cutting/coining (EVC^2^) processes for the manufacturing of various functional microstructure surfaces, a new type of two-degree-of-freedom (2-DOF) piezoelectrically actuated pseudo-decoupled compliant mechanism (PDCM) with non-orthogonal decoupling configurations was innovatively developed. First, the compliance model of the developed 2-DOF PDCM was theoretically built based on the finite beam-based matrix modeling (FBMM) and the matrix-based compliance modeling (MCM) method, then the effectiveness of the built compliance model was verified using the finite element analysis (FEA) method. Second, the dependencies of the trajectory tracking precision on the decoupling angle of the 2-DOF PDCM were investigated based on the popular FEA method, and the static and dynamic performances of the PDCM were also analyzed, such as the maximum stress and vibration modals. Third, a series of experiments was conducted on an established 2-DOF PDCM system to verify its main decoupling characteristics and dynamic performances. Finally, several critical conclusions on this study are briefly summarized as follows.

(a) In comparison to the FEA investigations, the built compliance model of the 2-DOF PDCM has acceptable modeling deviations in the primary motion and parasitic motion, namely, 8.7% and 5.2%, respectively, which mainly derive from the complex shear effects and stress concentration effects in the connections of different flexure modules. However, the built compliance model can effectively characterize the elastic deformation behaviors of developed 2-DOF PDCMs.

(b) A special elliptical (circular) trajectory is adopted to investigate the dependency of decoupling performances on the decoupling angle of the PDCM based on the FEA method, and the decoupling angle is optimally chosen to be 102.5° through the introduction of a dimensionless aspect ratio. From the FEA-based static and dynamic analyses, the maximum stress and the first modal frequency of the 2-DOF PDCM are 82.84 MPa and 4038.2 Hz, which can both satisfy the design requirements of the desired PDCM.

(c) The maximum and minimum errors between the experimental and FEA-based elliptical trajectories are 0.1331 μm and 0.0256 μm, respectively, which are only 5.3% and 1.0% with respect to the major semi-axis length of elliptical trajectory. Meanwhile, the experimental ellipse is larger than the FEA-based ellipse in different degrees due to the motion magnification of the 2-DOF PDCM. Moreover, the practical first-order and second-order resonant frequencies are 863 Hz and 1893 Hz, respectively, which are both significantly less than the FEA-simulated resonant frequencies because of the imperfect contacts and increasing motion inertia.

## Figures and Tables

**Table 1:** The dimension parameters and result comparisons for the 2-DOF PDCM.

**Dimension parameters**					
LSFM	*t*_1_ = 1 mm	*R*_1_ = 2.5 mm	*l*_1_ = 10 mm	*h*_1_ = 4 mm	*d*_1_ = 10 mm
RCFM	*t*_2_ = 1 mm	*R*_2_ = 3.0 mm	*l*_2_ = 10 mm	*h*_2_ = 4 mm	*d*_2_ = 7.0 mm
**Result analysis**					
		FBMM	FEA	Deviation	
*dx*/*Fx* or *dy*/*Fy*		1.15 × 10^-7^ m/N	1.26 × 10^-7^ m/N	8.7%	
*dy*/*Fx* or *dx*/*Fy*		1.45 × 10^-9^ m/N	1.53 × 10^-9^ m/N	5.2%	

**Table 2:** The experimental results and fitting elliptical parameters of the PDCM.

Motion parameters	Value	Elliptical parameters	Value
Amplitude in Y-axis /*A_y_*	2.5151 μm	Semi-major axis /*a*	2.5841 μm
Amplitude in Z-axis /*A_z_*	2.5132 μm	Semi-minor axis /*b*	2.4449 μm
Offset in Y-axis /*B_y_*	3.0000 μm	Center in Y-axis /*y_o_*	3.0012 μm
Offset in Z-axis /*B_z_*	2.9990 μm	Center in Z-axis /*z_o_*	2.9986 μm
-------	------	Inclined angle /*θ*	-44.9850°
